# Weight discrimination and eating disorder symptoms in early adolescence: a prospective cohort study

**DOI:** 10.1186/s40337-025-01404-w

**Published:** 2025-09-29

**Authors:** Jason M. Nagata, Arianna Thompson, Christiane K. Helmer, Kyle T. Ganson, Alexander Testa, Wesley R. Barnhart, Jinbo He, Fiona C. Baker, Jason M. Lavender

**Affiliations:** 1 Department of Pediatrics, University of California, San Francisco, 550 16th Street, 4th Floor, Box 0503, San Francisco, CA 94143 USA; 2 Factor-Inwentash Faculty of Social Work, University of Toronto, Toronto, 246 Bloor St W, Toronto, ON M5S 1V4 Canada; 3 Department of Management, Policy and Community Health, University of Texas Health Science Center at Houston, 7000 Fannin, Suite 1706, Houston, TX 77030 USA; 4 Department of Psychology, Suffolk University, 73 Tremont Street, 8th Floor, Boston, MA 02108 USA; 5 Department of Psychiatry, The University of North Carolina at Chapel Hill, 101 Manning Dr. #1, Chapel Hill, NC 27514 USA; 6 Department of Psychology, Bowling Green State University, 206 Psychology Building, Bowling Green, OH 43403 USA; 7 Department of Biosciences and BioinformaticsSchool of Science, Xi’an Jiaotong-Liverpool University, Suzhou, 215123 Jiangsu China; 8 Center for Health Sciences, SRI International, 333 Ravenswood Ave, Menlo Park, CA 94025 USA; 9 Military Cardiovascular Outcomes Research Program (MiCOR), Department of Medicine, Uniformed Services University of the Health Sciences, 4301 Jones Bridge Rd, Bethesda, MD 20814 USA; 10 The Metis Foundation, 84 NE Interstate 410 Loop # 325, San Antonio, TX 78216 USA

**Keywords:** Eating disorder, Disordered eating, Binge eating, Weight, Discrimination, Weight stigma, Adolescence

## Abstract

**Background:**

Weight discrimination is associated with adverse outcomes, including eating disorder (ED) symptoms, but few longitudinal studies have investigated this relationship in early adolescence. We examined the prospective association of weight discrimination with ED symptoms one year later in early adolescents, and the extent to which this association was moderated by body mass index (BMI) percentile and sex.

**Methods:**

We analyzed prospective data from Year 2 (2018–2020) and Year 3 (2019–2021) of the Adolescent Brain Cognitive Development (ABCD) Study (*N* = 9,079). To estimate the associations between self-reported experiences of weight discrimination in Year 2 and ED symptoms in Year 3, we conducted multiple logistic and ordinal logistic regression analyses, controlling for potential covariates, including ED symptoms in Year 2. Weight discrimination was measured using the Perceived Discrimination Scale. Presence of various ED symptoms was assessed via parent report using the Kiddie Schedule for Affective Disorders and Schizophrenia (KSADS-5). Participant BMI percentile and sex were also investigated as potential moderators.

**Results:**

Weight discrimination was prospectively associated with higher odds of worry about weight gain (adjusted odds ratio [aOR] 2.12, 95% confidence interval [CI] 1.08–4.14, *p* = 0.028), self-worth tied to weight (aOR 3.75, 95% CI 2.54–5.55, *p* < 0.001), inappropriate compensatory behaviors to prevent weight gain (aOR 2.75, 95% CI 2.02–3.74, *p* < 0.001), binge eating symptoms (aOR 1.72, 95% CI 1.10–2.68, *p* = 0.018), and distress about binge eating (aOR 2.26, 95% CI 1.33–3.85, *p* = 0.002) one year later. Weight discrimination was also associated with higher odds of a greater number of overall ED symptoms one year later (aOR 2.21, 95% CI 1.61–3.03, *p* < 0.001). A significant interaction by BMI percentile was also found: in adolescents with BMI of 5th to < 85th percentile, weight discrimination was more strongly and prospectively associated with higher odds of binge eating symptoms (aOR 3.32, 95% CI 1.27–8.68, *p* = 0.015) and binge eating distress (aOR 5.11, 95% CI 2.10–12.44, *p* < 0.001).

**Conclusions:**

Results support a prospective relationship between perceived weight discrimination and ED symptoms in early adolescents, and the differential associations based on BMI percentile highlight the need for interventions that address weight stigma across the weight spectrum.

**Supplementary Information:**

The online version contains supplementary material available at 10.1186/s40337-025-01404-w.

## Abstract (plain language summary)

Weight discrimination has been linked to negative health outcomes, including disordered eating and psychological distress, but there is a limited understanding of how this relationship impacts early adolescents. In this diverse, national sample of 9,079 adolescents aged 10–13 years, we found that those who experience weight discrimination were more likely to experience eating disorder symptoms such as tying their self-worth to weight gain and engaging in unhealthy behaviors to prevent weight gain, as well as experiencing a higher overall number of eating disorder symptoms, one year later. For adolescents with a BMI between the 5th and 85th percentiles, there was a stronger association of weight discrimination and binge eating behavior and distress about binge eating than for those with BMI above the 85th percentile. These findings highlight a prospective link between weight discrimination and eating disorder symptoms during early adolescence. Clinicians may help address these effects by screening for weight discrimination and disordered eating behaviors in adolescents of all body sizes and providing supportive, stigma-free care for all adolescents, regardless of BMI.

**Supplementary Information:**

The online version contains supplementary material available at 10.1186/s40337-025-01404-w.

## Background

Eating disorders (EDs) are serious mental health conditions that affect many adolescents, a phase of life defined by the World Health Organization as ages 10–19 [1]. Findings from recent systematic reviews suggest that the age-adjusted prevalence of a clinically diagnosed ED worldwide in adolescents and young adults is estimated to be around 0.3–5.7%, depending on sex and diagnostic criteria, with some countries approaching lifetime prevalences of 10% [2, 3]. Females are disproportionately affected by EDs, with studies in Western settings finding a prevalence of 16–18% by early adulthood [4–7]. Furthermore, evidence indicates that EDs in youth are becoming more common, particularly in the wake of the COVID-19 pandemic [8]. Disordered eating and ED symptoms (e.g., unhealthy weight control behaviors, fear of weight gain, overvaluation of shape/weight, disinhibited eating patterns) that do not meet the criteria for a diagnosis of a sub- or full-threshold ED are even more common, with one meta-analysis finding an overall prevalence of 22% for disordered eating behaviors in adolescents across 16 countries [9]. Moreover, such symptoms are associated with negative outcomes and elevated risk for developing full-threshold EDs [4, 10]. Importantly, EDs are associated with a range of adverse psychosocial and physical outcomes, such as cardiovascular complications, depression, and increased suicidality [11–15]. Furthermore, EDs are associated with an increased risk of mortality and have among the highest mortality rates among psychiatric disorders [15, 16]. A recent meta-analysis of mortality rates among those with EDs found up to a three-fold higher mortality rate in individuals with EDs compared to the general population, and up to a five-fold higher risk of death for those with anorexia nervosa (AN) [17]. Given the significant morbidity and mortality of EDs, understanding the factors contributing to ED development is essential for early intervention and prevention strategies.

One risk factor for ED psychopathology that has received increasing attention is weight discrimination, which has been defined as unjust and overt forms of weight-based prejudice and unfair treatment of individuals in larger bodies [18]. In children, this may take the form of weight-based teasing, a particular form of intentional physical, psychological, or social harassment [19]. Substantial proportions of adolescents experience forms of weight discrimination, with weight-based teasing reported among 15–34% of adolescents in the general population and weight discrimination reported by adolescents across all BMI categories [9, 19–23]. Weight-based teasing is even more prevalent in adolescents with larger bodies, with one study showing 63% of adolescents with a body mass index (BMI) ≥ 95th percentile reporting weight-based teasing and another indicating a similar prevalence among youth seeking weight loss [24, 25].

Notably, weight stigma and teasing have been found to be associated with disordered eating behaviors such as binge eating and body dissatisfaction, even when controlling for BMI and socioeconomic status, as well as numerous other adverse health outcomes, including psychological distress, depression, and metabolic syndrome [24, 26–28]. However, research has largely focused on older adolescents and adults, with a limited number of studies addressing weight discrimination in relation to EDs and ED symptoms in younger adolescents (ages 10–13). Given that early adolescence is a critical period for ED development (i.e., preceding the median age of ED onset at 12–15 years old) and that adequate nutrition is essential for growth and development during this developmental period, it is important to investigate weight discrimination in relation to ED psychopathology in younger populations [9, 29, 30].

Although cross-sectional findings in early adolescents have also suggested a correlation between weight teasing and disordered eating behaviors [22, 31–34], few studies have examined the prospective association of weight-based teasing with ED symptoms in this population [22, 31–34]. Findings from Project Eating and Activity over Time (EAT) have supported a prospective relationship between weight teasing in adolescents and disordered eating behavior, yet the study was limited to one geographic location and the mean age at study commencement was 14 [20, 28, 35]. A more recent prospective study found that among early adolescents (ages 10–11), weight discrimination was associated with an increased likelihood of meeting criteria for full-threshold or subthreshold EDs one year later [36]. However, this study did not examine the association between weight discrimination and specific ED symptoms, independent of the diagnosis. Another study of early adolescents (ages 8–12) also found that weight-based teasing at the individual level was indirectly associated with eating restraint and body dissatisfaction and that weight-based teasing at the classroom level was associated with eating restraint, although this study did not specifically examine other ED symptoms [37].

Although individuals from across the weight spectrum can experience weight-based stigma and discrimination, individuals with a higher BMI may be particularly at risk for such discrimination. For example, adolescents with BMI above the 85th percentile have been shown to have higher odds of experiencing weight discrimination [23]. Moreover, those with a larger body size may also be at greater risk for experiencing ED psychopathology. For instance, a higher BMI is prospectively and cross-sectionally associated with a higher risk for bulimia nervosa (BN) and disordered eating behaviors [9, 23, 38–40], as well as greater severity of ED symptoms, clinical impairment, and general psychopathology in individuals with EDs, excluding those with AN [41]. Moreover, in addition to sex-based differences in ED prevalence, data suggest that female adolescents may experience higher levels of weight stigma compared to their male counterparts, as well as a higher risk of weight bias internalization [42–45]. Given these findings, there is a need to better understand the extent to which BMI and sex may moderate the prospective association between weight discrimination and ED symptoms in youth.

Using data from the Adolescent Brain Cognitive Development (ABCD) Study, the aims of this study were to: (a) examine the prospective association of weight discrimination with ED symptoms in a national sample of early adolescents from the U.S., and (b) examine the extent to which this association was moderated by BMI and sex. This investigation builds upon prior investigations using ABCD data, including a study that found cross-sectional associations between weight discrimination and ED symptoms [33], and a prospective study that found that weight discrimination was associated with a greater likelihood of meeting criteria for full-threshold or subthreshold EDs at one-year follow-up [36]. However, neither study examined prospective associations of weight discrimination with individual ED symptoms, nor examined BMI or sex as potential moderators. In this study, we hypothesized that higher baseline weight discrimination would be associated with greater ED symptoms (e.g., worry about weight gain, self-worth tied to weight, inappropriate compensatory behaviors to prevent weight gain, binge eating symptoms, binge eating distress) one year later, and these prospective associations would be stronger among female adolescents and adolescents with a higher baseline BMI percentile.

## Methods

### Study sample

We analyzed prospective data from the ABCD Study, a longitudinal study of adolescent brain development and health in the U.S. At baseline (2016–2018), 11,875 children primarily aged 9–10 years were recruited from 21 demographically diverse sites across the U.S. using stratified, probability sampling. More details about the ABCD Study can be found elsewhere [46]. The study received institutional review board approval from the University of California, San Diego, and each of the 21 study sites. Participants provided written assent, while caregivers provided written informed consent. The data analyzed in this study were from the ABCD 5.1 release for Year 2 (2018–2020) and Year 3 (2019–2021), with the participants aged 10–13 years at Year 2 and 11–14 years at Year 3. Since youth-reported ED symptoms were only assessed in Year 2, we opted to use parent-reported ED symptoms to conduct prospective analyses. Of the participants enrolled in the study, 2,024 were missing parent-reported ED symptoms data, 585 were missing weight discrimination data, and 274 were missing sociodemographic data (Additional File 1: Appendix A). Our inclusion criteria resulted in a final analytic sample of 9,079 participants.

### Independent variable: weight discrimination (Year 2)

Weight discrimination was measured at Year 2 using the Perceived Discrimination Scale derived from the 2009 Boston Youth Survey [23, 47, 48]. Adolescents were asked, “In the past 12 months, have you felt people treated you unfairly or negatively: Because of your weight?” and responded with “Yes” or “No.”

### Dependent variable: eating disorder symptoms (Year 3)

ED symptoms were assessed via parent-report using the Kiddie Schedule for Affective Disorders and Schizophrenia (KSADS-5), a validated computerized tool for diagnostically categorizing child and adolescent mental health issues according to DSM-5 criteria [49]. In the ABCD Study, the KSADS-5 evaluated the frequency, duration, and characteristics of five ED symptoms: (1) worry about weight gain, (2) self-worth tied to weight, (3) inappropriate compensatory behaviors to prevent weight gain, (4) binge eating symptoms, and (5) distress about binge eating. To evaluate worry about weight gain, parents were asked, “In the past two weeks, how often has your child been preoccupied with gaining weight or worrying a lot about being fat?” Responses of “nearly every day” were coded as worrying about weight gain. Self-worth tied to weight was assessed with the question, “Does your child feel like his or her self-worth is tied to his or her weight?” Parents who responded “yes” were coded as endorsing this symptom. Parents were also asked about compensatory behaviors their child may have used to prevent weight gain, including eating foods with minimal calories, excessive exercise, vomiting, and using water pills, laxatives, or diet pills. Given the low prevalence of these behaviors in the non-clinical sample of this study, a single compensatory behavior variable was used reflecting the presence versus absence of any of the assessed behaviors. Binge eating symptoms were assessed with the question, “In the past two weeks, how often has your child had eating binges, when he or she lost control of his or her eating and ate way more than he or she needed, because your child was unable to stop himself or herself from eating?” A response of “yes” was coded as binge eating symptoms. Finally, distress about binge eating was assessed with the question, “How much discomfort or distress does binge eating cause your child?” Responses were rated on a Likert scale from 0 to 10, with the KSADS-5 classifying a response of 3 or higher as the cutoff to indicate distress about binge eating.

### Moderating variables: BMI percentile and sex

Body weight and height of each participant were measured by a trained research assistant. BMI was calculated as body weight (kg) divided by height squared (m²) and was used for summary statistics. To handle outliers, BMI values (kg/m²) were winsorized at the 1st and 99th percentiles based on Centers for Disease Control and Prevention (CDC) age- and sex-specific growth charts [50]. For interaction analyses, we converted raw BMI values to BMI percentiles and grouped them into the following four categories based on guidelines from the CDC, specific to age and sex: < 5th BMI percentile, 5th to < 85th BMI percentile, 85th to < 95th BMI percentile, and ≥ 95th BMI percentile [50]. Due to disruptions in data collection during the COVID-19 pandemic, we used the last observation carried forward method to impute missing data for BMI value and BMI percentile at Year 2, primarily imputing with Year 1 BMI data. Additionally, due to small cell sizes in our interaction analyses, BMI of < 5th percentile was removed, and the 85th to < 95th percentile and ≥ 95th percentile categories were combined into a single ≥ 85th percentile category. Another moderating variable assessed was sex assigned at birth, which was reported by parents/caregivers.

### Covariates

We adjusted for the following covariates (all derived from Year 2 data): age, sex (female or male), race and ethnicity (Asian, Black, Latino/Hispanic, Native American, White, or other), household income (grouped into six categories reflecting the U.S. median household income), highest parental education (high school or less vs. college education or more), and ABCD study site. These variables were chosen based on prior studies linking them to weight discrimination and/or ED symptoms [23, 51, 52]. Additionally, each respective ED symptom, as reported by the parent in Year 2, was included as a covariate.

### Statistical analysis

Data analyses were conducted using Stata 18 (StataCorp LLC). Descriptive statistics, including means, standard deviations, and percentages, were computed. Multiple logistic regression analyses were performed to estimate associations between weight discrimination in Year 2 as the independent variable and the presence (vs. absence) of ED symptoms in Year 3 (worry about weight gain, self-worth tied to weight, inappropriate compensatory behavior to prevent weight gain, binge eating, or distress about binge eating) as the dependent variable, adjusting for covariates, including the respective ED symptom in Year 2. Ordinal logistic regression analyses were also conducted to estimate associations between weight discrimination in Year 2 as the independent variable and the overall number of ED symptoms present at Year 3 (range: 0–5 ED symptoms) as the dependent variable, adjusting for covariates, including the overall number of ED symptoms present at Year 2. Regression model assumptions were met: multicollinearity was absent or low (VIFs < 2), the linearity of continuous predictors with log odds was supported (Box–Tidwell *p* > 0.05), observations were independent, and the sample size was adequate. For logistic regression, the outcome was binary, and no influential outliers were present (all Pregibon’s dbeta < 1.5). For ordinal logistic regression, the outcome was ordered, and the proportional odds assumption was met (Brant *p* > 0.05). Moderation analyses by BMI percentile (5th to < 85th percentile vs. ≥ 85th percentile) and sex (female vs. male) were conducted to assess how these factors influence the association between weight discrimination and ED symptoms, both for individual ED symptoms and overall number of ED symptoms. Sampling weights were applied to yield representative estimates based on the American Community Survey from the U.S. Census [53].

## Results

Table 1 reports the descriptive characteristics of the 9,079 adolescents included in our analytical sample, 48.8% of whom were female, and 44.2% of whom identified as a racial/ethnic minority. In Year 2, the mean BMI was 20.3 (± 4.3) kg/m², with a range of 13.3–33.2 kg/m², and the mean BMI percentile was 63.2 (± 30.4) with a range of 0.0–99.9. Out of our sample, 5.5% of participants reported experiencing weight discrimination (6.6% among < 5th BMI percentile, 2.5% among 5th to < 85th BMI percentile, 5.4% among 85th to < 95th BMI percentile, and 15.8% among 95th BMI percentile or higher). At Year 3, the presence of ED symptoms varied from 0.9% to 8.2% across specific symptoms.

**Table 1:** Sociodemographic characteristics and prevalence of weight discrimination and eating disorder symptoms in Adolescent Brain Cognitive Development (ABCD) Study participants (*N* = 9,079)

Sociodemographic characteristics (Year 2)	Mean (SD), range / %
Age (years)	12.0 (0.7), 10.6–13.8
Sex	
Female	48.8%
Male	51.2%
Race and ethnicity	
Asian	5.4%
Black	14.7%
Latino/Hispanic	19.4%
Native American	3.2%
Other^a^	1.4%
White	55.8%
Body mass index (BMI)	
BMI (kg/m²)^*b*^	20.3 (4.3), 13.3–33.2
BMI percentile	63.2 (30.4), 0.0–99.9
< 5th percentile	3.5%
5th to < 85th percentile	61.9%
85th to < 95th percentile	16.7%
95th percentile or higher	17.9%
Household income	
$24,999 or less	13.4%
$25,000 to $49,999	16.7%
$50,000 to $74,999	16.5%
$75,000 to $99,999	14.6%
$100,000 to $199,999	28.6%
$200,000 and greater	10.1%
Parent’s highest education	
High school education or less	11.3%
College education or more	88.7%
Weight discrimination (adolescent-reported)	5.5%
Eating disorder symptoms (parent-reported, Year 3)	
Worry about weight gain	0.9%
Self-worth tied to weight	4.1%
Inappropriate compensatory behaviors to prevent weight gain	8.2%
Binge eating symptoms	3.7%
Distress about binge eating	2.4%

ABCD sampling weights were applied to yield representative estimates based on the American Community Survey from the U.S. Census

^a^This subcategory was listed as “other” but with no specific racial and ethnic groups defined, although write-ins were allowed

^b^To handle outliers, BMI was winsorized at the 1st and 99th percentiles based on age- and sex-specific growth charts from the Centers for Disease Control and Prevention

Prospective associations between weight discrimination and ED symptoms are presented in Table 2. After covariate adjustment, we found that weight discrimination was prospectively associated with higher odds of worry about weight gain (adjusted odds ratio [aOR] 2.12, 95% confidence interval [CI] 1.08–4.14, *p* = 0.028), self-worth tied to weight (aOR 3.75, 95% CI 2.54–5.55, *p* < 0.001), inappropriate compensatory behaviors to prevent weight gain (aOR 2.75, 95% CI 2.02–3.74, *p* < 0.001), binge eating symptoms (aOR 1.72, 95% CI 1.10–2.68, *p* = 0.018), and distress about binge eating (aOR 2.26, 95% CI 1.33–3.85, *p* = 0.002) one year later. Weight discrimination was associated with a 2.21 (95% CI 1.61–3.03, *p* < 0.001) higher odds of experiencing a greater number of overall ED symptoms one year later.

**Table 2:** Prospective associations between weight discrimination and eating disorder symptoms in the Adolescent Brain Cognitive Development (ABCD) Study (*N* = 9,079)

Eating disorder symptoms (Year 3, dependent variable)	Weight discrimination (Year 2, independent variable)			
	Unadjusted OR (95% CI)	p	Adjusted OR (95% CI)^a^	p
Worry about weight gain	**3.75 (1.99**,** 7.07)**	**< 0.001**	**2.12 (1.08**,** 4.14)**	**0.028**
Self-worth tied to weight	**4.68 (3.37**,** 6.50)**	**< 0.001**	**3.75 (2.54**,** 5.55)**	**< 0.001**
Inappropriate compensatory behaviors to prevent weight gain	**3.10 (2.35**,** 4.09)**	**< 0.001**	**2.75 (2.02**,** 3.74)**	**< 0.001**
Binge eating symptoms	**3.00 (2.07**,** 4.34)**	**< 0.001**	**1.72 (1.10**,** 2.68)**	**0.018**
Distress about binge eating	**3.60 (2.33**,** 5.55)**	**< 0.001**	**2.26 (1.33**,** 3.85)**	**0.002**
Overall number of eating disorder symptoms	**3.29 (2.56**,** 4.23)**	**< 0.001**	**2.21 (1.61**,** 3.03)**	**< 0.001**

Bold indicates *p* < 0.05. Models represent the abbreviated output from 10 logistic regression models and 2 ordinal logistic regression models (overall number of eating disorder symptoms) with weight discrimination in Year 2 as the independent variable and eating disorder symptoms in Year 3 as the dependent variable. ABCD sampling weights were applied to yield representative estimates based on the American Community Survey from the U.S. Census

^a^Adjusted for eating disorder symptoms at Year 2, age, sex, race and ethnicity, household income, parent education, and study site

Table 3 shows prospective BMI percentile-stratified analyses examining associations of weight discrimination with ED symptoms. There was a significant interaction between BMI percentile (5th to < 85th percentile vs. 85th or higher) and weight discrimination for worry about weight gain (*p* = 0.007), binge eating symptoms (*p* = 0.020), and distress about binge eating (*p* = 0.010). Although weight discrimination was significant with worry about weight gain in the overall analytic sample, this association was not statistically significant within the BMI-stratified groups of 5th to < 85th percentile and 85th percentile or higher, yet the strength of the association differed between the two BMI groups (*p* = 0.007). In adolescents with BMI of 5th to < 85th percentile, presence of weight discrimination was prospectively associated with higher odds of binge eating symptoms (aOR 3.32, 95% CI 1.27–8.68, *p* = 0.015) and binge eating distress (aOR 5.11, 95% CI 2.10–12.44, *p* < 0.001) one year later, after adjusting for covariates, while these associations were not significant for BMI percentile of 85th or higher. There was no significant interaction between sex and weight discrimination in relation to any individual ED symptom or the overall number of ED symptoms.

**Table 3:** Prospective associations between weight discrimination and eating disorder symptoms in the Adolescent Brain Cognitive Development (ABCD) Study, stratified by BMI percentile

Eating disorder symptoms (Year 3, dependent variable)	Weight discrimination (Year 2, independent variable)				
	BMI of 5th to < 85th percentile		BMI of 85th percentile or higher		
	Adjusted OR (95% CI)^a^	p	Adjusted OR (95% CI)^a^	p	p for interactions
Worry about weight gain	3.47 (0.95, 12.63)	0.059	1.36 (0.59, 3.14)	0.469	**0.007**
Self-worth tied to weight	**2.67 (1.20**,** 5.90)**	**0.016**	**2.83 (1.80**,** 4.45)**	**< 0.001**	0.949
Inappropriate compensatory behaviors to prevent weight gain	**3.05 (1.64**,** 5.68)**	**< 0.001**	**1.94 (1.37**,** 2.75)**	**< 0.001**	0.249
Binge eating symptoms	**3.32 (1.27**,** 8.68)**	**0.015**	1.17 (0.73, 1.87)	0.523	**0.020**
Distress about binge eating	**5.11 (2.10**,** 12.44)**	**< 0.001**	1.42 (0.80, 2.52)	0.230	**0.010**
Overall number of eating disorder symptoms	**2.81 (1.59**,** 4.96)**	**< 0.001**	**1.64 (1.15**,** 2.33)**	**0.006**	0.122

Bold indicates *p* < 0.05. Models represent the abbreviated output from 10 logistic regression models and 2 ordinal logistic regression models (overall number of eating disorder symptoms) with weight discrimination in Year 2 as the independent variable and eating disorder symptoms in Year 3 as the dependent variable. ABCD sampling weights were applied to yield representative estimates based on the American Community Survey from the U.S. Census

^a^Adjusted for eating disorder symptoms at Year 2, age, sex, race and ethnicity, household income, parent education, and study site

## Discussion

In a diverse cohort of early U.S. adolescents, this study found that those who experienced weight discrimination had a significantly greater odds of experiencing certain ED symptoms one year later, including worry about weight gain, self-worth tied to weight, inappropriate compensatory behaviors, binge eating symptoms, and distress about binge eating. Participants who reported weight discrimination were also more likely to experience a greater overall number of ED symptoms. Our findings are largely consistent with those of prior investigations using ABCD data, which found that weight discrimination was cross-sectionally associated with greater odds of binge eating, compensatory vomiting, fear of weight gain, and self-worth tied to weight [33], and prospectively associated with a greater likelihood of meeting study criteria for a sub- or full-threshold ED one year later [36].

In the current investigation, the strongest adjusted association indicated that experiencing weight discrimination was prospectively associated with a nearly four times greater odds of self-worth being tied to weight, consistent with previous findings that the internalization of weight stigma may play a critical role in the development of ED psychopathology [32, 54, 55]. This pattern is well-aligned with theoretical models such as Tomiyama’s Cyclic Obesity/Weight-Based Stigma (COBWEBS) model [56] and Corrigan’s self-stigma model [57], both of which highlight how exposure to weight-based stigma, particularly when internalized, can result in increased psychological distress and maladaptive eating behaviors. This finding is particularly salient in the context of risk for developing a full-threshold ED, given that overvaluation of body weight/shape (i.e., an excessive influence of weight/shape on how one judges oneself) is conceptualized as a key underlying risk/maintenance factor for ED psychopathology [58]. Moreover, the presence of weight discrimination was prospectively associated with a nearly three times greater odds of engaging in any inappropriate compensatory behavior to prevent weight gain, further highlighting the potentially harmful psychological and behavioral impacts of experiencing weight discrimination during early adolescence.

In contrast to the hypotheses, moderation analyses revealed no significant interactions based on sex, and for BMI, the significant interactions that were found were opposite from expected. The lack of differences in the association between weight discrimination and ED symptoms across sex may be due to the early age range of the sample, given that physical and socioemotional differences become more prominent during and after pubertal onset, as well as the role that sex-based differences in pubertal onset play in the development of EDs [45, 59]. For BMI, adolescents with a 5th to < 85th percentile BMI had *greater* odds of experiencing binge eating symptoms and distress about binge eating compared to their peers with a BMI at or above the 85th percentile, despite this latter group being more likely to face societal weight stigma [60]. Weight discrimination was associated with a more than threefold greater odds of binge eating symptoms and five-fold greater odds of distress about binge eating in the 5th to < 85th percentile BMI group. The significant interaction found suggests that weight discrimination may have a particularly deleterious impact on adolescents who may not expect to be stigmatized based on their weight, potentially leading to increased psychological distress and thus increased disordered eating behaviors [61]. Consistent with this notion, prior research has suggested that individuals with a lower BMI who experience weight stigma may perceive this as incongruent with societal expectations, leading to increased emotional distress and subsequent maladaptive eating behaviors [60]. It is also possible that adolescents with higher BMI percentiles, who have been shown to experience greater levels of internalized weight bias, may have already developed different coping mechanisms or resilience factors compared to those who do not anticipate such discrimination [62]. Additionally, it is important to consider other factors, such as co-occurring psychopathology and body dissatisfaction, that may differ between the two BMI groups and potentially influence the perception of weight discrimination and the development of binge eating symptoms and distress. Nevertheless, weight discrimination was significantly associated with greater odds of self-worth tied to weight, inappropriate compensatory behaviors, and the overall number of ED symptoms for both BMI percentile groups, reinforcing the potentially harmful consequences of such discrimination for early adolescents across the weight spectrum.

Overall, these findings highlight the prospective relationship of weight discrimination and ED symptoms in both male and female young adolescents of all body sizes, indicating the need for interventions that address the impact of weight stigma across those of all body sizes. Relevant frameworks such as COBWEBS and the self-stigma model underscore the importance of addressing not only overt discrimination, but also the internalized beliefs and social messages that may reinforce eating pathology. Public health and policy efforts should emphasize reducing internalized weight stigma and weight-based discrimination across contexts, including schools and healthcare settings, to mitigate adverse psychological and behavioral consequences. Clinically, providers should be aware of the potential harms of weight discrimination, even in adolescents who fall within a “normal” BMI range and should adopt weight-inclusive approaches that prioritize overall well-being rather than weight status alone. Additional interventions such as early education about weight stigma during healthcare training, equipping healthcare facilities with size-inclusive equipment (e.g., appropriately sized waiting room chairs and exam tables), and screening for and addressing internalized weight bias in both patients and healthcare providers should also be implemented to reduce weight-based discrimination in healthcare settings [63–65].

### Strengths and limitations

While this study provides valuable insight into the prospective relationships between weight discrimination and ED symptoms in early U.S. adolescents, it has several limitations. First, the question used to assess weight discrimination did not provide specific examples of being treated unfairly or negatively due to weight. This lack of clarity may have made it difficult for adolescents to fully understand the question, potentially contributing to underreporting. Additionally, this question was a single, non-validated item. However, it was drawn from the widely used Perceived Discrimination Scale, which has shown strong psychometric validity in adolescent populations and has been highly correlated with mental health in a large national sample across diverse ages, races, and socioeconomic backgrounds [48, 66, 67]. Furthermore, this scale was originally derived from the 2009 Boston Youth Survey, which demonstrated a high co-occurrence of discrimination and bullying [47]. Second, the analysis relied on parent-reported assessments of adolescents’ ED symptoms. Parental reports were used due to data availability at both Year 2 and 3, which was necessary for the prospective design; however, this may not fully capture adolescents’ internal or subjective experiences [68]. Prior research using Year 2 data from the ABCD Study has documented discordance between parent and adolescent reports of ED symptoms [69]. Some symptoms (e.g., binge eating or compensatory behaviors to lose weight) may be more frequently reported by adolescents due to their private or internalized nature and others may be better identified by parents, possibly due to adolescents’ denial of the problem or lack of insight [70–72]. Thus, future studies utilizing adolescent self-reports of both weight discrimination and ED symptoms are warranted. Additionally, while this study design assessed prospective relationships, we cannot investigate causality. As with any longitudinal study, participant attrition could introduce bias if certain groups were more likely to drop out between Year 2 and Year 3. This may particularly affect adolescents experiencing severe ED symptoms or greater psychosocial distress, leading to an underestimation of the true relationships [73].

Though BMI percentiles are commonly used in pediatric research, they do not capture differences in body composition, which could influence experiences of weight discrimination [64]. We categorized BMI into 5th to < 85th percentile and 85th percentile or higher due to an insufficient sample size to examine associations separately for adolescents with a BMI below the 5th percentile or between the 85th and 95th percentiles. While this generally allowed for sufficient statistical power, it may have obscured more nuanced differences between these groups. Additionally, results for “worry about weight gain” after stratifying by BMI group may have been underpowered, as the prevalence of this ED symptom for the overall sample was only 0.9%. Another limitation of this study was that sex was restricted to binary options, which fails to capture intersex experiences and, importantly, sex is not synonymous with gender identity. A lack of inclusion of diverse gender identities may have led to the exclusion or misclassification of nonbinary and gender diverse participants, a population known to be at higher risk for ED psychopathology [74, 75]. Finally, the variables analyzed were largely dichotomous, focusing on absence versus presence of weight discrimination experiences and ED symptoms. Future studies would benefit from measures that account for severity and frequency of experiences weight discrimination and symptoms, as well as the type and source of discrimination. This would allow for further understanding of the prospective associations identified in this study and help identify key areas for targeted intervention against weight discrimination.

## Conclusion

Despite these limitations, our findings indicate that weight discrimination in early adolescence is a significant prospective predictor of a variety of ED symptoms. Future research could explore potential mediators (e.g., weight stigma internalization, negative affect) to identify pathways linking weight discrimination to ED symptoms. Overall, these results indicate a relationship between weight discrimination and some key ED symptoms in male and female young adolescents across body sizes, and future interventions aimed at reducing weight stigma to prevent adverse mental health outcomes for all adolescents are recommended.

## Supplementary Information

Below is the link to the electronic supplementary material.

Supplementary Material 1. Title of data: Appendix A. Description of data: Comparison of the sociodemographic characteristics of the Adolescent Brain Cognitive Development (ABCD) study participants included vs. excluded in the analysis.

## Data Availability

Data used in the preparation of this article were obtained from the ABCD Study ( [https://abcdstudy.org](https:/abcdstudy.org) ), held in the NIMH Data Archive (NDA).

## Abbreviations

ABCD: Adolescent Brain Cognitive Development Study
ED: eating disorder
AN: anorexia nervosa
BN: bulimia nervosa
BMI: body mass index
