# Supplementary material for: Weight discrimination and eating disorder symptoms in early adolescence: a prospective cohort study
Source: J Eat Disord. 2025 Sep 29;13:216. doi: 10.1186/s40337-025-01404-w (PMC12482715; doi:10.1186/s40337-025-01404-w)
Supplement: Supplementary file 1 — Supplementary Material 1. Title of data: Appendix A. Description of data: Comparison of the sociodemographic characteristics of the Adolescent Brain Cognitive Development (ABCD) study participants included vs. excluded in the analysis. [file 40337_2025_1404_MOESM1_ESM.docx]

| Appendix A. Comparison of the sociodemographic characteristics of the Adolescent Brain Cognitive Development (ABCD) study participants included vs. excluded in the analysis | | | |
| --- | --- | --- | --- |
| Sociodemographic characteristics | Included (n=9,079) | Excluded (n=2,883) | p |
| Age (years) | 12.0 (0.7) | 12.1 (0.7) | 0.002 |
| Sex (%) |  |  | 0.969 |
| Female | 48.8% | 48.9% |  |
| Male | 51.2% | 51.1% |  |
| Race and ethnicity (%) |  |  | <0.001 |
| Asian | 5.4% | 5.8% |  |
| Black | 14.7% | 25.7% |  |
| Latino/Hispanic | 19.4% | 22.2% |  |
| Native American | 3.2% | 3.0% |  |
| Other | 1.4% | 1.6% |  |
| White | 55.8% | 41.7% |  |
| Body mass index |  |  | <0.001 |
| <5th percentile | 3.5% | 3.4% |  |
| 5th to <85th percentile | 61.9% | 55.1% |  |
| 85th to <95th percentile | 16.7% | 18.0% |  |
| 95th percentile or higher | 17.9% | 23.5% |  |
| Household income (%) |  |  | <0.001 |
| $24,999 or less | 13.4% | 21.3% |  |
| $25,000 to $49,999 | 16.7% | 22.5% |  |
| $50,000 to $74,999 | 16.5% | 14.1% |  |
| $75,000 to $99,999 | 14.6% | 9.6% |  |
| $100,000 to $199,999 | 28.6% | 26.6% |  |
| $200,000 or greater | 10.1% | 6.0% |  |
| Parent's highest education (%) |  |  | <0.001 |
| High school education or less | 11.3% | 27.0% |  |
| College education or more | 88.7% | 73.0% |  |
